# Methano­ldinitrato[*N*-(2-pyridylmethyl­ene)aniline]copper(II)

**DOI:** 10.1107/S1600536809041075

**Published:** 2009-10-17

**Authors:** Young-Inn Kim, Hong Woo Lee, Ji-Hoon Kim, Sun Young Park, Sung Kwon Kang

**Affiliations:** a Department of Chemistry Education, Interdisciplinary Program of Advanced Information and Display Materials, and Center for Plastic Information Systems, Pusan National University, Busan 609-735, Republic of Korea; b Department of Chemistry, Chungnam National University, Daejeon 305-764, Republic of Korea

## Abstract

The Cu atom in the title compound, [Cu(NO_3_)_2_(C_12_H_10_N_2_)(CH_3_OH)], adopts a square-pyramidal geometry, being ligated by two N atoms of the bidentate *N*-(2-pyridylmethyl­ene)­aniline (ppma) ligand, two O atoms of NO_3_ ligands and one O atom of a methanol molecule, which occupies the apical position. The phenyl ring on the ppma ligand is twisted out of the pyridine plane, forming a dihedral angle of 42.9 (1)°. In the crystal, inter­molecular O—H⋯O hydrogen bonds between methanol and NO_3_ ligands form an extensive one-dimensional network extending parallel to [100].

## Related literature

For general background on magnetic materials, see: Lu *et al.* (2007 ▶); Mukherjee *et al.* (2008 ▶); Tao *et al.* (2004 ▶). For related structures, see: Lee *et al.* (2008 ▶); Addison *et al.* (1984 ▶). For general background on electron paramagnetic resonance spectra, see: Mohapatra *et al.* (2008 ▶).
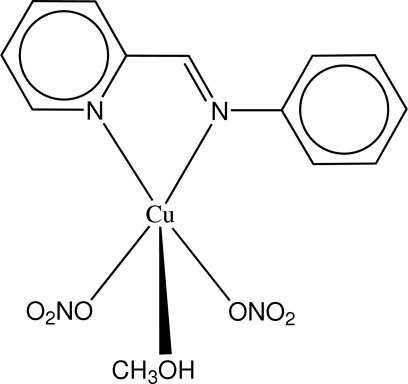


## Experimental

### 

#### Crystal data

[Cu(NO_3_)_2_(C_12_H_10_N_2_)(CH_4_O)]
*M* *_r_* = 401.82
Orthorhombic, 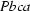

*a* = 14.5924 (13) Å
*b* = 13.4826 (12) Å
*c* = 17.0060 (13) Å
*V* = 3345.8 (5) Å^3^
*Z* = 8
Mo *K*α radiation
μ = 1.35 mm^−1^
*T* = 295 K
0.20 × 0.18 × 0.14 mm

#### Data collection

Bruker SMART CCD area-detector diffractometer
Absorption correction: multi-scan (*SADABS*; Bruker, 2002 ▶) *T*_min_ = 0.76, *T*_max_ = 0.823
17118 measured reflections
3289 independent reflections
2098 reflections with *I* > 2σ(*I*)
*R*_int_ = 0.037

#### Refinement

*R*[*F* ^2^ > 2σ(*F* ^2^)] = 0.038
*wR*(*F* ^2^) = 0.101
*S* = 1.03
3289 reflections
229 parameters
H atoms treated by a mixture of independent and constrained refinement
Δρ_max_ = 0.43 e Å^−3^
Δρ_min_ = −0.34 e Å^−3^

### 

Data collection: *SMART* (Bruker, 2002 ▶); cell refinement: *SAINT* (Bruker, 2002 ▶); data reduction: *SAINT*; program(s) used to solve structure: *SHELXS97* (Sheldrick, 2008 ▶); program(s) used to refine structure: *SHELXL97* (Sheldrick, 2008 ▶); molecular graphics: *ORTEP-3 for Windows* (Farrugia, 1997 ▶); software used to prepare material for publication: *WinGX* (Farrugia, 1999 ▶).

## Supplementary Material

Crystal structure: contains datablocks global, I. DOI: 10.1107/S1600536809041075/jh2108sup1.cif

Structure factors: contains datablocks I. DOI: 10.1107/S1600536809041075/jh2108Isup2.hkl

Additional supplementary materials:  crystallographic information; 3D view; checkCIF report

## Figures and Tables

**Table 1:** Hydrogen-bond geometry (Å, °)

*D*—H⋯*A*	*D*—H	H⋯*A*	*D*⋯*A*	*D*—H⋯*A*
O23—H23⋯O18^i^	0.69 (3)	2.15 (3)	2.817 (4)	162 (4)

Symmetry code: (i) 
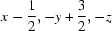
.

**Fig. 1.**
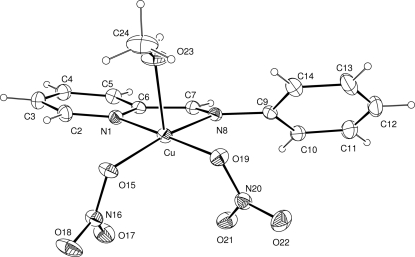
Molecular structure of (I), showing the atom-numbering scheme and 30% probability ellipsoids.

## supplementary crystallographic information

### Comment

Schiff base complexes of transition metal complexes have great importance over the years due to their versatility of the steric and electronic properties and their possible applications as molecular based magnetic materials (Lu *et al.*, 2007; Mukherjee *et al.*, 2008; Tao *et al.*, 2004). As a part of this research, we reported copper halides complexes with N2 bidentate Schiff base ligand derived from 2-pyridinecarboxylaldehyde and benzylamine(Lee *et al.*, 2008), in which the reaction of copper(II) chloride leads to a dimeric complex whereas copper(II) bromide affords a monomeric copper complex. In this study, we reacted copper(II) nitrate with the similar Schiff base in methanol and prepared a monomeric penta-coordinated copper(II) complex, Cu(ppma)(NO_3_)_2_(CH_3_OH) (I).

In the title compound, the Cu atom adopts a square pyramidal geometry, being ligated by two N atoms of the bidentate *N*-(2-pyridylmethylene)aniline (ppma) ligand, two O atoms of NO3 ligands, and one O atom of methanol which occupies the apical position. The angles around Cu atom at the basal position are in the range of 80.8 (1) - 96.6 (1)°. The calculated trigonality index, τ = 0.12, indicates that the Cu atom is in an almost square pyramidal geometry (Addison *et al.*, 1984). The phenyl ring on the ppma ligand is twisted out of the pyridine plane, and forms a dihedral angle of 42.9 (1) °. The intermolecular O23—H23—O18^i^ [symmetry code: (i) *x* - 1/2, -*y* + 3/2, -*z*] hydrogen bond allows to form an extensive one-dimensional network, which stabilizes the crystal structure.

EPR (electron paramagnetic resonance) spectra of I compound were obtained both for solid and for frozen glass samples (toluene/methanol) at 77 K. The powder EPR spectrum exhibits isotropic feature, <g>=2.151. The solution EPR spectrum exhibits well defined hyperfine structure with parallel and perpendicular components, g(parallel) = 2.328, g(perpendicular) = 2.065 and A(parallel) =142x10^-4^ cm^-1^, typically indicating a d*_x_*^2^_-_*_y_*^2^ ground state, g(parallel) > g(perpendicular) > 2.0023 (Mohapatra *et al.*, 2008). The magnetic susceptibilities of the title compound were collected as a function of temperatures (4 - 300 K). The magnetic susceptibility data increases as the temperatures decrease exhibiting a paramagnetic behavior. Magnetic susceptibility data follows the Curie-Weiss law showing the features of a discrete monomeric complex. A linear regression results in a Curie-Weiss temperature θ = 0.55 K and a Curie constant C = 0.45 cm^3^ K mol^-1^.

### Experimental

*N*-(2-pyridylmethylene)aniline was synthesized from the direct reaction of 2-pyridinecarboxyaldehyde and aniline. 2-Pyridinecarboxyaldehyde (2 mmol) dissolved in 20 ml of absolute methanol was added dropwise to a methanolic solution of aniline (2 mmol) and then refluxed overnight. After cooling to room temperature, a solution of Cu(NO_3_)_2_ 3H_2_O (2 mmol) in 20 ml of absolute methanol was added to the mixed solution of 2-pyridinecarboxyaldehyde and aniline (ppma solution). The solution was changed to dark green color immediately. The resulting solution was allowed to stand at room temperature. The green crystals were obtained by slow evaporation in methanol.

### Refinement

The H23 atom was located in a difference map and refined freely with O—H = 0.69 (3) Å. Other H atoms were positioned geometrically and refined using a riding model, with C—H = 0.93 - 0.96 Å, and with *U*_iso_(H) = 1.2*U*_eq_(C) for aromatic and 1.5*U*_eq_(C) for methyl H atoms.

### Crystal data

**Table:**

[Cu(NO_3_)_2_(C_12_H_10_N_2_)(CH_4_O)]	*F*(000) = 1640
*M**_r_* = 401.82	*D*_x_ = 1.595 Mg m^−^^3^
Orthorhombic, *P**b**c**a*	Mo *K*α radiation, λ = 0.71073 Å
Hall symbol: -P 2ac 2ab	Cell parameters from 4394 reflections
*a* = 14.5924 (13) Å	θ = 2.4–22.8°
*b* = 13.4826 (12) Å	µ = 1.35 mm^−^^1^
*c* = 17.0060 (13) Å	*T* = 295 K
*V* = 3345.8 (5) Å^3^	Block, green
*Z* = 8	0.2 × 0.18 × 0.14 mm

### Data collection

**Table:**

Bruker SMART CCD area-detector diffractometer	2098 reflections with *I* > 2σ(*I*)
φ and ω scans	*R*_int_ = 0.037
Absorption correction: multi-scan (*SADABS*; Bruker, 2002)	θ_max_ = 26°, θ_min_ = 2.4°
*T*_min_ = 0.76, *T*_max_ = 0.823	*h* = −11→18
17118 measured reflections	*k* = −10→16
3289 independent reflections	*l* = −20→14

### Refinement

**Table:**

Refinement on *F*^2^	0 restraints
Least-squares matrix: full	H atoms treated by a mixture of independent and constrained refinement
*R*[*F*^2^ > 2σ(*F*^2^)] = 0.038	*w* = 1/[σ^2^(*F*_o_^2^) + (0.0421*P*)^2^ + 1.1597*P*] where *P* = (*F*_o_^2^ + 2*F*_c_^2^)/3
*wR*(*F*^2^) = 0.101	(Δ/σ)_max_ < 0.001
*S* = 1.03	Δρ_max_ = 0.43 e Å^−^^3^
3289 reflections	Δρ_min_ = −0.34 e Å^−^^3^
229 parameters	

### Special details

**Table:**

Geometry. All e.s.d.'s (except the e.s.d. in the dihedral angle between two l.s. planes) are estimated using the full covariance matrix. The cell e.s.d.'s are taken into account individually in the estimation of e.s.d.'s in distances, angles and torsion angles; correlations between e.s.d.'s in cell parameters are only used when they are defined by crystal symmetry. An approximate (isotropic) treatment of cell e.s.d.'s is used for estimating e.s.d.'s involving l.s. planes.

### Fractional atomic coordinates and isotropic or equivalent isotropic displacement parameters (Å^2^)

**Table:**

	*x*	*y*	*z*	*U*_iso_*/*U*_eq_	
Cu	0.22734 (3)	0.80769 (3)	0.05883 (2)	0.04304 (16)	
N1	0.19359 (18)	0.8656 (2)	−0.04406 (14)	0.0443 (7)	
C2	0.2115 (2)	0.8304 (3)	−0.1155 (2)	0.0562 (9)	
H2	0.2447	0.7718	−0.1198	0.067*	
C3	0.1832 (3)	0.8766 (3)	−0.1831 (2)	0.0630 (10)	
H3	0.1961	0.8489	−0.2319	0.076*	
C4	0.1354 (3)	0.9642 (3)	−0.1777 (2)	0.0611 (10)	
H4	0.116	0.9971	−0.2228	0.073*	
C5	0.1169 (2)	1.0027 (2)	−0.10368 (19)	0.0529 (9)	
H5	0.0859	1.0626	−0.0983	0.063*	
C6	0.1449 (2)	0.9511 (2)	−0.03880 (18)	0.0416 (8)	
C7	0.1207 (2)	0.9783 (2)	0.04160 (18)	0.0438 (8)	
H7	0.0898	1.0371	0.0522	0.053*	
N8	0.14329 (17)	0.91931 (18)	0.09677 (14)	0.0423 (6)	
C9	0.1175 (2)	0.9408 (2)	0.17667 (18)	0.0466 (8)	
C10	0.1211 (2)	1.0360 (3)	0.2067 (2)	0.0627 (10)	
H10	0.1417	1.0883	0.1758	0.075*	
C11	0.0938 (3)	1.0521 (4)	0.2833 (3)	0.0858 (14)	
H11	0.0948	1.1161	0.3038	0.103*	
C12	0.0653 (3)	0.9751 (5)	0.3291 (3)	0.0951 (16)	
H12	0.0486	0.9867	0.3811	0.114*	
C13	0.0611 (3)	0.8808 (4)	0.2995 (2)	0.0864 (14)	
H13	0.0404	0.8291	0.331	0.104*	
C14	0.0876 (2)	0.8620 (3)	0.2226 (2)	0.0650 (10)	
H14	0.0854	0.798	0.2022	0.078*	
O15	0.32671 (16)	0.71875 (15)	0.01603 (14)	0.0542 (6)	
N16	0.4040 (2)	0.7588 (2)	0.00143 (16)	0.0540 (7)	
O17	0.40926 (18)	0.8491 (2)	−0.00598 (15)	0.0781 (7)	
O18	0.47131 (18)	0.70493 (19)	−0.0054 (2)	0.0903 (10)	
O19	0.26151 (16)	0.75883 (17)	0.16300 (13)	0.0561 (6)	
N20	0.3146 (2)	0.8210 (2)	0.19904 (18)	0.0565 (8)	
O21	0.33689 (17)	0.89636 (19)	0.16336 (14)	0.0714 (8)	
O22	0.3405 (2)	0.80158 (19)	0.26547 (15)	0.0846 (9)	
O23	0.1281 (2)	0.6864 (2)	0.0490 (2)	0.0773 (10)	
H23	0.084 (2)	0.704 (3)	0.042 (2)	0.049 (13)*	
C24	0.1395 (3)	0.5872 (3)	0.0572 (3)	0.0998 (16)	
H13A	0.0819	0.5543	0.0492	0.15*	
H13B	0.1616	0.5729	0.1092	0.15*	
H13C	0.183	0.5639	0.0191	0.15*	

### Atomic displacement parameters (Å^2^)

**Table:**

	*U*^11^	*U*^22^	*U*^33^	*U*^12^	*U*^13^	*U*^23^
Cu	0.0416 (3)	0.0385 (3)	0.0489 (2)	0.00253 (18)	0.00262 (18)	0.00149 (17)
N1	0.0430 (17)	0.0432 (17)	0.0469 (16)	0.0001 (13)	0.0024 (12)	−0.0009 (12)
C2	0.058 (3)	0.055 (2)	0.056 (2)	0.0043 (18)	0.0079 (18)	−0.0077 (18)
C3	0.070 (3)	0.075 (3)	0.044 (2)	−0.004 (2)	0.0028 (19)	−0.0035 (19)
C4	0.067 (3)	0.070 (3)	0.047 (2)	−0.008 (2)	−0.0028 (18)	0.0109 (18)
C5	0.054 (2)	0.048 (2)	0.057 (2)	0.0029 (17)	−0.0011 (18)	0.0079 (17)
C6	0.0360 (19)	0.0396 (19)	0.0491 (19)	−0.0035 (16)	0.0025 (14)	0.0018 (15)
C7	0.040 (2)	0.0379 (19)	0.053 (2)	0.0028 (16)	0.0016 (15)	−0.0022 (15)
N8	0.0393 (17)	0.0423 (16)	0.0452 (15)	−0.0030 (13)	0.0036 (12)	−0.0031 (12)
C9	0.0339 (19)	0.057 (2)	0.0483 (18)	0.0038 (16)	0.0032 (15)	−0.0020 (17)
C10	0.052 (2)	0.072 (3)	0.064 (2)	−0.003 (2)	0.0058 (19)	−0.020 (2)
C11	0.069 (3)	0.113 (4)	0.075 (3)	0.004 (3)	0.008 (2)	−0.038 (3)
C12	0.068 (3)	0.163 (5)	0.054 (3)	0.020 (3)	0.010 (2)	−0.021 (3)
C13	0.069 (3)	0.128 (4)	0.062 (3)	0.016 (3)	0.022 (2)	0.023 (3)
C14	0.060 (3)	0.072 (3)	0.062 (2)	0.006 (2)	0.011 (2)	0.007 (2)
O15	0.0380 (14)	0.0448 (14)	0.0799 (17)	−0.0012 (11)	0.0119 (12)	0.0028 (11)
N16	0.048 (2)	0.049 (2)	0.0647 (18)	−0.0012 (18)	0.0070 (15)	0.0009 (15)
O17	0.078 (2)	0.0534 (16)	0.103	−0.0108 (15)	0.0194 (16)	0.0031 (15)
O18	0.0405 (17)	0.0652 (19)	0.165 (3)	0.0090 (14)	0.0224 (18)	−0.0068 (17)
O19	0.0627 (16)	0.0481 (15)	0.0577 (14)	−0.0049 (13)	−0.0082 (12)	0.0056 (12)
N20	0.050 (2)	0.067 (2)	0.0519 (18)	0.0023 (16)	0.0013 (16)	0.0093 (17)
O21	0.072 (2)	0.0728 (18)	0.0689 (16)	−0.0241 (15)	−0.0123 (14)	0.0210 (14)
O22	0.100 (2)	0.098 (2)	0.0556 (16)	−0.0161 (16)	−0.0205 (15)	0.0193 (14)
O23	0.0456 (19)	0.0467 (18)	0.140 (3)	0.0003 (15)	−0.0207 (18)	0.0095 (15)
C24	0.071 (3)	0.050 (3)	0.179 (5)	−0.009 (2)	−0.013 (3)	0.019 (3)

### Geometric parameters (Å, °)

**Table:**

Cu—O19	1.955 (2)	C10—C11	1.378 (5)
Cu—N1	1.979 (2)	C10—H10	0.93
Cu—O15	2.017 (2)	C11—C12	1.365 (6)
Cu—N8	2.046 (2)	C11—H11	0.93
Cu—O23	2.191 (3)	C12—C13	1.369 (6)
N1—C2	1.330 (4)	C12—H12	0.93
N1—C6	1.356 (4)	C13—C14	1.388 (5)
C2—C3	1.370 (5)	C13—H13	0.93
C2—H2	0.93	C14—H14	0.93
C3—C4	1.375 (5)	O15—N16	1.274 (3)
C3—H3	0.93	N16—O17	1.226 (3)
C4—C5	1.388 (4)	N16—O18	1.227 (3)
C4—H4	0.93	O19—N20	1.295 (3)
C5—C6	1.367 (4)	N20—O22	1.220 (3)
C5—H5	0.93	N20—O21	1.228 (3)
C6—C7	1.459 (4)	O23—C24	1.355 (4)
C7—N8	1.273 (4)	O23—H23	0.69 (3)
C7—H7	0.93	C24—H13A	0.96
N8—C9	1.439 (4)	C24—H13B	0.96
C9—C10	1.382 (4)	C24—H13C	0.96
C9—C14	1.389 (4)		
			
O19—Cu—N1	176.44 (10)	C10—C9—N8	121.8 (3)
O19—Cu—O15	86.75 (9)	C14—C9—N8	117.3 (3)
N1—Cu—O15	95.43 (10)	C11—C10—C9	119.0 (4)
O19—Cu—N8	96.60 (10)	C11—C10—H10	120.5
N1—Cu—N8	80.75 (10)	C9—C10—H10	120.5
O15—Cu—N8	169.09 (10)	C12—C11—C10	120.5 (4)
O19—Cu—O23	89.20 (11)	C12—C11—H11	119.8
N1—Cu—O23	93.60 (11)	C10—C11—H11	119.8
O15—Cu—O23	90.21 (11)	C11—C12—C13	120.7 (4)
N8—Cu—O23	100.20 (11)	C11—C12—H12	119.7
C2—N1—C6	117.8 (3)	C13—C12—H12	119.7
C2—N1—Cu	128.2 (2)	C12—C13—C14	120.3 (4)
C6—N1—Cu	114.0 (2)	C12—C13—H13	119.9
N1—C2—C3	123.0 (3)	C14—C13—H13	119.9
N1—C2—H2	118.5	C13—C14—C9	118.5 (4)
C3—C2—H2	118.5	C13—C14—H14	120.7
C2—C3—C4	119.1 (3)	C9—C14—H14	120.7
C2—C3—H3	120.4	N16—O15—Cu	117.0 (2)
C4—C3—H3	120.4	O17—N16—O18	121.8 (3)
C3—C4—C5	118.7 (3)	O17—N16—O15	119.7 (3)
C3—C4—H4	120.7	O18—N16—O15	118.4 (3)
C5—C4—H4	120.7	N20—O19—Cu	111.31 (19)
C6—C5—C4	118.9 (3)	O22—N20—O21	123.6 (3)
C6—C5—H5	120.5	O22—N20—O19	119.0 (3)
C4—C5—H5	120.5	O21—N20—O19	117.4 (3)
N1—C6—C5	122.4 (3)	C24—O23—Cu	130.4 (3)
N1—C6—C7	113.7 (3)	C24—O23—H23	118 (3)
C5—C6—C7	123.8 (3)	Cu—O23—H23	112 (3)
N8—C7—C6	118.1 (3)	O23—C24—H13A	109.5
N8—C7—H7	121	O23—C24—H13B	109.5
C6—C7—H7	121	H13A—C24—H13B	109.5
C7—N8—C9	120.1 (3)	O23—C24—H13C	109.5
C7—N8—Cu	112.5 (2)	H13A—C24—H13C	109.5
C9—N8—Cu	127.1 (2)	H13B—C24—H13C	109.5
C10—C9—C14	121.0 (3)		

### Hydrogen-bond geometry (Å, °)

**Table:**

*D*—H···*A*	*D*—H	H···*A*	*D*···*A*	*D*—H···*A*
O23—H23···O18^i^	0.69 (3)	2.15 (3)	2.817 (4)	162 (4)

Symmetry codes: (i) *x*−1/2, −*y*+3/2, −*z*.
